# Assisted reproductive technology and association with childhood cancer subtypes

**DOI:** 10.1002/cam4.5114

**Published:** 2022-08-05

**Authors:** Natalie B. Gulrajani, Samuel Montes, Daniel McGough, Courtney E. Wimberly, Ameera Khattab, Eleanor C. Semmes, Lisa Towry, Jennifer L. Cohen, Jillian H. Hurst, Daniel Landi, Sherika N. Hill, Kyle M. Walsh

**Affiliations:** ^1^ Children's Health and Discovery Institute, Department of Pediatrics Duke University School of Medicine Durham North Carolina USA; ^2^ Master of Biomedical Sciences Program Duke University School of Medicine Durham North Carolina USA; ^3^ Department of Neurosurgery and Preston Robert Tisch Brain Tumor Center Duke University School of Medicine Durham North Carolina USA; ^4^ My Childhood Cancer Program Alex's Lemonade Stand Foundation Bala Cynwyd Pennsylvania USA; ^5^ Division of Medical Genetics, Department of Pediatrics Duke University School of Medicine Durham North Carolina USA; ^6^ Frank Porter Graham Child Development Institute The University of North Carolina Chapel Hill North Carolina USA; ^7^ Duke Cancer Institute Duke University School of Medicine Durham North Carolina USA

**Keywords:** assisted reproductive technology, cancer risk factors, epidemiology, in vitro fertilization, liver cancer, osteosarcoma, pediatric cancer

## Abstract

**Objectives:**

To investigate the association between assisted reproductive technology (ART) use and childhood cancer subtype.

**Study Design:**

We deployed a cross‐sectional survey of 1701 parents of children with cancer about their ART use, demographics, and gestational and perinatal factors. Multivariable logistic regression modeled the association between ART use, birthweight and multiple gestation status with childhood cancer, by subtype.

**Results:**

ART use was highest among children with osteosarcoma relative to children with other cancer types, and this association was statistically significant in multivariable models (OR = 4.4; 95% CI = 1.7–11.3; *p* = 0.0020). ART use was also elevated among children with hepatoblastoma, but this relationship appeared to be due to the strong associations between ART use and lower birthweight in our sample. No specific ART modality appeared to drive these associations. In univariate models, multiple gestation was associated with a 2.7‐fold increased odds of hepatoblastoma (OR = 2.71; 95% CI = 1.14–6.42; *p* = 0.02) and a 1.6‐fold increased odds of neuroblastoma (OR = 1.62; 95% CI = 1.03–2.54; *p* = 0.03), but these associations were not retained in multivariable models.

**Conclusions:**

Associations between ART use and hepatoblastoma risk may be attributable to birthweight, a known hepatoblastoma risk factor. ART use may also be associated with osteosarcoma, independent of birthweight, an association not previously observed in studies limited to cancers diagnosed before adolescence. Evaluating long‐term health outcomes in children conceived by ART, throughout adolescence and potentially into adulthood, appears warranted.

## Graphical abstract

Epidemiologic data suggest an increase in U.S. childhood cancer incidence overall, though rates vary across cancer subtypes and few modifiable risk factors for childhood cancer have been identified. A cross‐sectional survey of 1701 parents of children with cancer revealed associations between use of assisted reproductive technology and risk of childhood hepatoblastoma and osteosarcoma. the latter of which has not been previously observed in studies limited to cancers before adolescence. Evaluating long‐term health outcomes in children conceived by ART, throughout adolescence and potentially into adulthood, appears warranted.

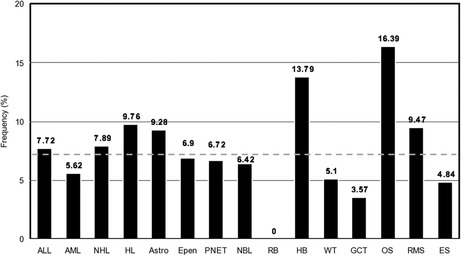

## 1 INTRODUCTION

Cancer is a leading cause of death in children ages 5–14 years.^1^ While mortality rates have decreased and 5‐year survival rates exceed 80%, nearly 2000 childhood cancer‐associated deaths occur annually in the U.S.^2^ Epidemiologic data suggest a modest increase in childhood cancer incidence, although the factors underlying this trend remain largely unresolved.^3^ One possibility is that the prevalence of underlying risk factors may be changing. In the U.S., the incidence of acute lymphoblastic leukemia (ALL) is highest in the Hispanic population, and as this population has grown, national rates of ALL have risen accordingly.^4^ Whether increased cancer incidence is also driven by modifiable risk factors merits further evaluation.

While childhood cancer incidence has increased overall, this pattern does not hold across all subtypes. For example, rates of hepatoblastoma and central nervous system (CNS) tumors increased substantially from 1992–2004.^3^ The increase in CNS tumor incidence is partially attributable to incidental detection of indolent tumors with the expanded use of medical imaging^5^; however, factors underlying increases in hepatoblastoma incidence remain unclear. Additionally, trends in childhood cancer incidence differ by race and ethnicity, suggesting non‐uniform changes in the prevalence of underlying risk factors across populations.^6^

Although numerous genetic factors increase childhood cancer risk, relatively few modifiable risk factors have been conclusively identified. Several pre‐ and perinatal factors have been associated with childhood cancer risk, including in utero exposure to ionizing radiation,^7^ very high or very low birthweight,^8^ in utero diethylstilbestrol exposure,^9^ and congenital cytomegalovirus (CMV) infection.^10^, ^11^ Such findings suggest that small perturbations to cellular differentiation during gestation may have outsized effects on both normal and malignant cellular development.

The use of assisted reproductive technologies (ART) to conceive is increasingly common – accounting for 1.9% of U.S. births in 2017 – and has previously been associated with elevated childhood cancer risk.^12^, ^13^, ^14^, ^15^ Because ART is a broad term that includes many types of infertility treatment, including in‐vitro fertilization (IVF), use of donor sperm or eggs, intracytoplasmic sperm injection (ICSI), gamete intrafallopian transplantation (GIFT), intrauterine insemination (IUI), frozen embryo transfer (FET), and fertility drugs, each ART method could have substantially different impacts on in utero development. ART use has been associated with reduced birthweight, increased birthweight (in the case of FET), multiple gestations, a sex‐ratio skewed toward male births, and increased occurrence of congenital/developmental abnormalities.^16^, ^17^, ^18^, ^19^, ^20^ These observations indicate that ART may alter the typical course of fetal development, with potentially different effects across ART modalities. Given the potential public health impact of increased risk of childhood cancer, further investigation of ART use and its association with specific cancer subtypes is warranted.

To evaluate the association of ART with childhood subtypes, we performed a cross‐sectional analysis of childhood cancer patients from Alex's Lemonade Stand Foundation's *My Childhood Cancer: Survey Series* cohort. Parents representing >1700 childhood cancer patients provided data on ART utilization, multiple gestation status, birthweight, and other demographic and pre/perinatal factors. We sought to examine whether ART use confers greater risk of specific childhood cancer subtypes, accounting for contributions from other prenatal/perinatal factors.

## 2 METHODS

### 2.1 Study population

To explore associations between ART use and childhood cancers, we have partnered with Alex's Lemonade Stand Foundation (ALSF) to conduct an ongoing series of longitudinal surveys of families affected by childhood cancer. Initiated in 2011, the ALSF *My Childhood Cancer* (*MCC*): *Survey Series* explores families' experiences and attitudes from diagnosis, throughout treatment and follow‐up care, and after bereavement (when applicable).^21^ The English‐language survey is publicly hosted on the Alex's Lemonade Stand Foundation webpage and advertised through Facebook, Twitter, and ALSF's childhood cancer‐specific listserv. Parents are eligible to complete the survey if they have a child (living or deceased) who was diagnosed with cancer prior to the child's eighteenth birthday. Parents that navigate to the MCC survey complete a registration form with contact and basic demographic information, then are contacted by email to complete future surveys – including the diagnosis survey. Parents can complete surveys in one sitting or may return to it at a later time within 30 days of survey initiation. To date, 3150 families have participated in the *MCC* survey series. In this cross‐sectional study, we examined responses to the ALSF MCC diagnosis survey completed between August 2012 and April 2019, as prior versions of the survey did not collect data on ART use. Thus, analyses for this study were performed on the subgroup with available ART data (*N* = 1701 respondents), limiting to one parental respondent per family. Median time from diagnosis to survey completion was 2 years.

### 2.2 Survey instruments

Childhood cancer type and patient/parental demographics are collected at *MCC: Survey Series* registration. Survey respondents were asked “Did [child]’s biological mother receive any medical help in order to become pregnant with [child]?” Responses of “Yes” and “No” were collapsed into an indicator for ART use. Those who responded “Not sure” were excluded. Those who indicated that they received medical help to conceive were asked to answer whether any or all of the following methods of ART were used: IVF, fertility drugs, donor sperm/donor eggs, ICSI, IUI, and GIFT. At the time of data analysis, FET was not a specified ART modality in the survey, but has since been added. We assume that any respondents whose child was conceived via FET would have answered “Yes” to the ART question, and may have additionally endorsed additional ART modalities (e.g., “fertility drugs”). The “Yes” and “No” answers to these questions along with the “No” answers to any ART use were collapsed into binary indicators for each ART modality. Respondents who reported use of more than one ART type were included in each specific ART type variable used, but were counted only once in the overall ART use variable. Respondents (94% U.S.‐based) recorded child's birthweight in categories to the nearest pound in the following bins: “3 pounds or less” (≤1.80 kg), “4–5 pounds” (1.81–2.72 kg), “6–9 pounds” (2.73–4.53 kg), “10–11 pounds” (4.54–5.44 kg), “12 pounds or more” (≥5.45 kg), and “Not sure”. Birthweight was modeled as an ordinal variable. A question about whether the child with cancer was a singleton or multiple gestation was collapsed into an indicator for multiparity. Respondent race/ethnicity was collapsed into an indicator for “non‐Hispanic white” versus “American Indian/Alaskan Native,” “Native Hawaiian/Pacific Islander,” “Black/African American,” “Other race,” and “Hispanic or Latino (of any race).” Household income was recorded in the following bins: <$20,000; $20,000–$49,999; $50,000–$74,999; $75,000–$99,999; $100,000–$149,999; ≥$150,000 and modeled as an ordinal variable. Median time from diagnosis to survey completion was 2 years and did not differ across cancer subtype.

Dependent variables were specific childhood cancer subtype compared to all other subtypes collapsed, including: Hodgkin's lymphoma, non‐Hodgkin's lymphoma, germ cell tumor, Kidney/Wilms' tumor, hepatoblastoma/liver cancer, neuroblastoma, retinoblastoma, rhabdomyosarcoma, osteosarcoma, Ewing sarcoma, an “all sarcomas” subgroup, ALL, and acute myeloid leukemia (AML). We also included three brain/spinal tumor subgroups: primitive neuroectodermal tumors (PNETs) including medulloblastoma, supratentorial PNETs, and atypical teratoid/rhabdoid tumor (AT/RT); glioma, including astrocytoma/anaplastic astrocytoma, juvenile pilocytic astrocytoma (JPA), diffuse intrinsic pontine glioma (DIPG), glioblastoma; and ependymoma.

### 2.3 Statistical analyses

Independent variables of interest in this analysis included ART use, ART type, birthweight, and multiple gestation status. Survey respondent's race/ethnicity, household income, prematurity (birth prior to 37 weeks gestation), presence of other siblings, delivery mode (vaginal vs. Cesarean), maternal age, child's birth year, and years between child's birth and survey completion were included in models as potential confounders. We also included child's sex in models because it could potentially act as a confounder (e.g., boys generally have higher birthweight than girls and male children are at increased risk of CNS tumors)^22^ or as an effect modifier (which was evaluated using interaction terms).

Relationships between independent and dependent variables were assessed using chi‐square tests for independence or *t*‐tests for difference. Fisher's exact tests were used when analyzing specific ART types due to small sample sizes. For multivariate analyses, logistic regression was used to examine potential relationships between variables and to control for potential confounders. Our study design, in which a specific cancer subtype is compared to all other childhood cancer subtypes pooled, can reduce differential recall bias since all surveyed parents had a child diagnosed with cancer. Missing data rates for modeled covariates were very low (5% or less for all covariates). If data were missing for a modeled covariate, that individual was excluded from the model. For all statistical tests, alpha = 0.05 was used to determine statistical significance. Stata version 16.0 was used for data analysis.

## 3 RESULTS

**FIGURE 1:**
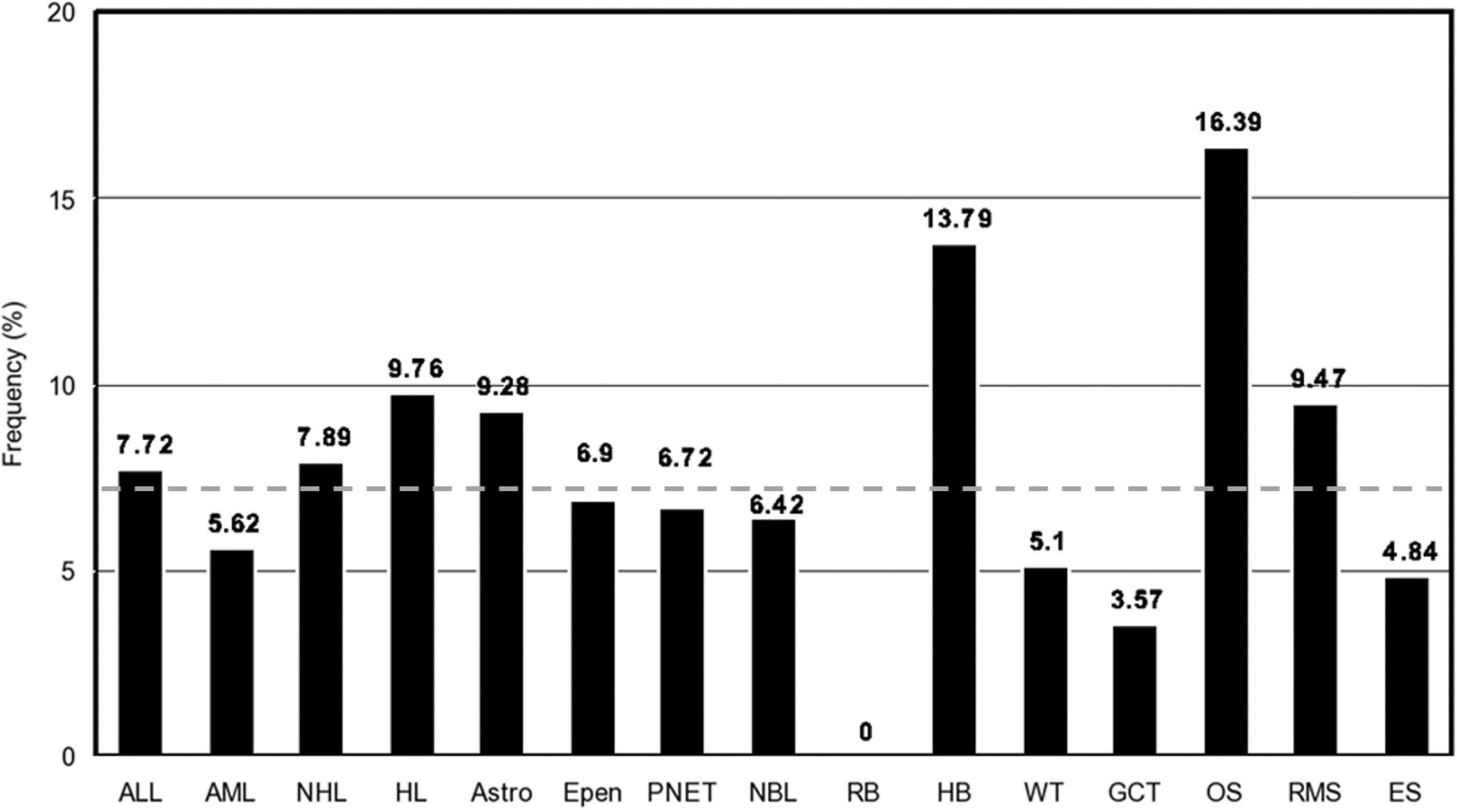
Proportion of survey respondents' children, by cancer type, who were conceived with use of Assisted Reproductive Technology in a cross‐sectional survey of participants enrolled in the *My Childhood Cancer Survey Series*. The dashed horizontal line indicates the rate of ART usage across all cancer types, combined (7.4%). Cancer types, from left to right, are: Acute lymphoblastic leukemia (ALL, *N* = 492), Acute myeloid leukemia (AML, *N* = 89), non‐Hodgkin lymphoma (NHL, *N* = 76), Hodgkin lymphoma (HL, *N* = 41), Astrocytoma (Astro, *N* = 97), Ependymoma (Epen, *N* = 29), primitive neuroectodermal tumors including medulloblastoma, AT/RT and supratentorial PNETs (PNET, *N* = 119), Neuroblastoma (NBL, *N* = 187), Retinoblastoma (RB, *N* = 28), Hepatoblastoma (HB, *N* = 29), Wilms tumor (WT, *N* = 98), Germ cell tumors (GCT, *N* = 28), Osteosarcoma (OS, *N* = 55), Rhabdomyosarcoma (RMS, *N* = 95), Ewing sarcoma (ES, *N* = 62).

Between August 2012 and April 2019, a total of 1701 respondents from unique families completed the diagnosis survey asking about use of ART to conceive. Median time from diagnosis to survey completion was 2 years (IQR = 1–7). Respondents were largely female, the biologic parent, and identified as non‐Hispanic white. The majority of children were 6–9 pounds at birth (2.72–4.53 kg), singletons, and had siblings at the time of diagnosis (Table 1). 126 respondents (7.4%) reported using ART to become pregnant, of which 85% specified the type of ART and 36% reported using more than one form of ART. Parental ART use was most frequent among children with osteosarcoma (16%), followed by hepatoblastoma (14%), and was least common among children with germ cell tumors (4%) and retinoblastoma (0%) (Figure 1). The sex‐ratios within most cancer subtypes in the sample were consistent with population‐based estimates. The exceptions included non‐significant, inverse associations between male sex and several cancer subtypes where population‐based data show a male preponderance: osteosarcoma, AML, and ependymoma.

**TABLE 1:** Demographic characteristics of survey respondents

	Number of respondents (*n* = 1701)	Proportion of study population or mean (SD)
Respondent biologic parent	1673	0.98
Respondent gender		
Female	1589	0.93
Male	111	0.07
Missing	1	<0.01
Respondent race/ethnicity		
Non‐Hispanic White	1582	0.93
Other	105	0.06
Missing	14	0.01
Birthweight		
1.80 kg or fewer	26	0.02
1.81–2.71 kg	107	0.06
2.72–4.53 kg	1468	0.86
4.54–5.44 kg	92	0.05
≥5.45 kg	5	<0.01
Missing	3	<0.01
Household income at diagnosis		
Less than $20,000	129	0.08
$20,000–$49,999	368	0.22
$50,000–$74,999	365	0.21
$75,000–$99,999	313	0.18
$100,000–$149,999	254	0.15
$150,000+	180	0.11
Missing	92	0.05
Child Sex		
Female	933	0.55
Male	767	0.45
Missing	1	<0.01
ART use^a^		
Any ART	126	0.07
IVF	27	0.02
Fertility drugs	103	0.06
Donor Sperm/Eggs	10	0.01
ICSI	13	0.01
IUI	24	0.01
GIFT	1	<0.01
Missing	18	0.01
Child age at diagnosis	…	6.25 (4.97)
0–2 years	408	0.24
3–5 years	317	0.19
6–9 years	459	0.27
10+ years	487	0.29
Missing	30	0.02
Birth period		
Before 1990	85	0.05
1990–1994	110	0.06
1995–1999	281	0.17
2000–2004	418	0.25
2005–2009	430	0.25
2010 and later	363	0.21
Missing	14	0.01
Cancer subtype		
Hodgkin lymphoma	41	0.02
Non‐Hodgkin lymphoma	76	0.04
Germ cell tumor	28	0.02
Wilm's tumor	98	0.06
Hepatoblastoma	29	0.02
Neuroblastoma	187	0.11
Retinoblastoma	28	0.02
Rhabdomyosarcoma	95	0.06
Osteosarcoma	55	0.03
Ewing sarcoma	62	0.04
All sarcomas	250	0.15
ALL	492	0.29
AML	89	0.05
PNET	119	0.07
Ependymoma	29	0.02
Astrocytoma	97	0.06
Other	138	0.08
Siblings		
None	384	0.23
1+	1301	0.76
Missing	16	0.01
Multiparity		
Singleton	1625	0.96
Multiple gestation	68	0.04
Missing	8	<0.01

Abbreviations: ART, assisted reproductive technology; GIFT, gamete intrafallopian transfer; ICSI, intracytoplasmic sperm injection; IUI, intrauterine insemination; IVF, in vitro fertilization.

^a^ Respondents could select multiple types of ART, if applicable.

Univariate relationships between cancer subtype and ART use, multiparity, and birthweight were tested (Table S1), comparing specific cancer subtypes to all other subtypes collapsed. ART use was associated with a 2.6‐fold increased odds of osteosarcoma relative to all other cancer types (OR = 2.56; 95% CI = 1.22–5.35; *p* = 0.01) and a 2‐fold increased odds of hepatoblastoma (OR = 2.03; 95% CI = 0.70–5.93; *p* = 0.19), although this latter association did not reach statistical significance. Multiple gestation was associated with a 2.7‐fold increased odds of hepatoblastoma (OR = 2.71; 95% CI = 1.14–6.42; *p* = 0.02) and a 1.6‐fold increased odds of neuroblastoma (OR = 1.62; 95% CI = 1.03–2.54; *p* = 0.03). Children with hepatoblastoma had significantly lower birthweight than children with other cancer types (*p* < 0.001), while children with germ cell tumors had modestly higher birthweight than children with other cancer types (*p* = 0.08) (Table S1).

In the entire sample, ART use was associated with lower birthweight (*p* < 0.001) and with being a multiple gestation (*p* < 0.001). Multiple gestations were also associated with lower birthweight (*p* < 0.001). To disentangle these associations, we compared each childhood cancer subtype to all other cancer subtypes collapsed, and report childhood cancer subtype associations for ART use, multiple gestation status, and birthweight from multivariable models additionally adjusted for child sex, race/ethnicity, household income, years between birth and survey completion, gestational age, presence of siblings, delivery mode, maternal age, and child's birth year. In this multivariable model, the association between ART use and osteosarcoma persisted (OR = 4.4; 95% CI = 1.7–11.3; *p* = 0.0020). For hepatoblastoma, however, the magnitude of effect from univariate analyses (OR = 2.03) was strongly attenuated in the multivariable model (OR = 1.6; 95% CI = 0.38–6.7; *p* = 0.52) (Table 2). Univariate associations between multiple gestation status and both hepatoblastoma and neuroblastoma were attenuated in multivariable models, as were the associations between low birthweight and hepatoblastoma and higher birthweight and germ cell tumors (Table 2). Inclusion of a “sex*ART”, “sex*birthweight”, or “sex*multiple gestation status” interaction term did not reveal any significant interactions with child sex.

**TABLE 2:** Relationships between ART use, multiparous birth, birthweight and cancer subtype^a^

Cancer Type	ART use (OR^b^, 95% CI)	*p*‐value	Multiparous birth (OR^b^, 95% CI)	*p*‐value	Birthweight^c^ (OR^b^, 95% CI)	*p*‐value
Hodgkin lymphoma (*N* = 41)	0.90 (0.19, 4.3)	0.90	1.5 (0.25, 8.9)	0.67	0.44 (0.16, 1.2)	0.12
Non‐Hodgkin lymphoma (*N* = 76)	0.89 (0.26, 3.0)	0.84	NA	NA	0.84 (0.38, 1.9)	0.67
Germ cell tumor (*N* = 28)	NA	NA	NA	NA	2.1 (0.64, 7.0)	0.22
Wilm's tumor (*N* = 98)	0.55 (0.16, 1.9)	0.34	1.3 (0.32, 5.0)	0.74	1.1 (0.53, 2.1)	0.88
Hepatoblastoma (*N* = 29)	1.6 (0.38, 6.7)	0.52	2.9 (0.54, 15.3)	0.22	0.64 (0.23, 1.8)	0.41
Neuroblastoma (*N* = 187)	0.73 (0.33, 1.6)	0.44	1.6 (0.54, 4.5)	0.42	1.4 (0.86, 2.3)	0.18
Retinoblastoma (*N* = 28)	NA	NA	NA	NA	0.50 (0.10, 2.5)	0.39
Rhabdomyosarcoma (*N* = 95)	1.6 (0.67, 3.8)	0.30	1.23 (0.31, 4.9)	0.77	1.15 (0.58, 2.3)	0.69
Osteosarcoma (*N* = 55)	**4.4 (1.7, 11.3)**	**0.0020**	0.99 (0.11, 8.9)	0.99	1.2 (0.46, 3.1)	0.72
Ewing sarcoma (*N* = 62)	1.1 (0.32, 3.7)	0.90	0.56 (0.06, 5.0)	0.60	0.94 (0.40, 2.3)	0.90
All sarcomas (*N* = 250)	1.8 (0.99, 3.1)	0.053	1.5 (0.63, 3.6)	0.36	1.1 (0.69, 1.7)	0.76
ALL (*N* = 492)	0.93 (0.34, 2.6)	0.89	0.38 (0.092, 1.5)	0.18	0.78 (0.39, 1.5)	0.47
AML (*N* = 89)	1.1 (0.34, 3.4)	0.91	0.36 (0.039, 3.4)	0.37	0.78 (0.36, 1.7)	0.53
PNET^d^ (*N* = 119)	1.1 (0.46, 2.7)	0.81	0.63 (0.13, 3.0)	0.56	1.1 (0.61, 2.1)	0.69
Ependymoma (*N* = 29)	0.62 (0.076, 5.1)	0.66	1.54 (0.15, 16.0)	0.72	0.90 (0.26, 3.1)	0.87
Astrocytoma^e^ (*N* = 97)	1.8 (0.80, 4.0)	0.16	1.0 (0.22, 4.9)	0.97	1.6 (0.86, 3.0)	0.13

*p*‐values <0.05 appear in bold. Abbreviations: ART, assisted reproductive technology; ALL, acute lymphoblastic leukemia; AML, acute myeloid leukemia; PNET, primitive neuroectodermal tumor.

^a^ Multivariable logistic regression, including: ART use, multiparity, birthweight, child sex, race/ethnicity, household income, years between birth and survey completion, gestational age, presence of siblings, delivery mode, maternal age, and child's birth year.

^b^ Odds ratios by subtype, comparing the listed cancer subtype to all other childhood cancer patients in the study. Therefore, it should be noted that the reference group changes slightly for each subtype comparison.

^c^ Birthweight was recorded in the following categories and modeled as an ordinal variable: ≤1.80 kg, 1.81–2.71 kg, 2.72–4.53 kg, 4.54–5.44 kg, ≥5.45 kg.

^d^ Including medulloblastoma and AT/RT.

^e^ Including pilocytic astrocytoma, glioblastoma, and DIPG.

To isolate the effect of specific ART modality on cancer subtype, additional analyses were conducted for osteosarcoma and hepatoblastoma, which had the highest univariate odds ratios with ART use (Table S2). Every ART modality was similarly positively associated with hepatoblastoma and osteosarcoma, although no association reached statistical significance due to the limited sample size for each ART modality. Overall, our results did not identify any particular ART modality that appeared to drive the overall association of ART use with hepatoblastoma and osteosarcoma.

## 4 DISCUSSION

ART is an increasingly common method to overcome infertility, but perinatal risks associated with its use continue to raise concerns. The proportion of ART‐assisted conceptions leading to births in the United States were first recorded in 1996, and have more than tripled since then, accounting for 1.9% of all live‐births in the US in 2017.^15^ Associations between ART use and birthweight, a skewed sex‐ratio, congenital/developmental abnormalities, and potential associations with childhood cancer risk suggest that ART may alter typical fetal development.^12^, ^13^, ^14^, ^16^, ^17^, ^18^, ^19^ We observed an association between ART use and childhood osteosarcoma in our analyses, independent of birthweight, multiple gestation status, and other potential confounders. We also observed modest evidence of association between ART use and hepatoblastoma, potentially confounded by the strong association between low birthweight and hepatoblastoma risk. Univariate associations between higher birthweight and increased risk of neuroblastoma and germ cell tumors were also observed, aligned with previous registry‐based reports,^8^, ^23^, ^24^ but did not persist in multivariable models.

Although associations between ART and childhood cancer risk have been studied previously, subtype‐stratified risk and risk beyond the first decade of life remain unclear.^25^ Williams, et al. conducted a population‐based study of all children born in Britain from 1992–2008 and observed an increased risk of hepatoblastoma and rhabdomyosarcoma in association with ART use. The study assessed potential moderating and confounding factors, and attributed hepatoblastoma risk primarily to low birthweight in association with ART use.^26^ More recently, Spector, et al. observed that hepatoblastoma risk was significantly associated with IVF use, but they did not include birthweight in their modeling.^13^ Although they did not identify associations between ART use and osteosarcoma risk, the follow‐up time after birth was approximately 8 years, making it unlikely that adolescent‐onset cancers (i.e., osteosarcoma) would be captured. Williams, et al. also found no association between ART use and osteosarcoma risk, though only a small proportion of children were followed‐up at 15 years‐old and the average follow‐up time was approximately 8 years.^26^ Although osteosarcoma risk is associated with taller adolescent and adult stature, recent registry studies have not observed an association between osteosarcoma risk and infant birth length or birthweight.^8^, ^27^, ^28^ Our analyses support the independence of osteosarcoma risk and birthweight, but implicate ART in osteosarcomagenesis, independent of sex, birthweight and multiple gestation status.

In addition to its association with birthweight and multiple gestations, ART use has also been associated with a sex‐ratio skewed toward male births and an increased incidence of congenital/developmental abnormalities.^16^, ^17^, ^18^, ^19^ A recent murine study identified impaired imprinting of the inactivated X chromosome as a primary epigenetic barrier for female embryo development that was responsible for sex‐skewing toward male births following IVF.^29^ Such dysregulated epigenetic programming could lead to overexpression of key genes located in imprinted regions, both on the X chromosome and the autosomes, providing a potential molecular mechanism for these ART‐associated phenotypes. Importantly, in murine models this epigenetic dysregulation was resolved through retinoic acid supplementation, providing a potential opportunity for risk interception.^29^

Hepatoblastoma is believed to arise from primary hepatoblasts and even less differentiated hepatic stem cells/fetal liver multi‐potent progenitor cells. These highly proliferative cells can differentiate into a variety of tissues, including hepatocytes, bile ducts, and bone.^30^ Furthermore, 40% of HB tumors have mixed histology, including both epithelial and mesenchymal elements. These multipotential stem cells are likely reliant on local spatiotemporal cues, and may therefore be particularly susceptible to epigenetic dysregulation of adjacent cell types and microenvironmental transcripts. While the osteosarcoma cell of origin remains an open question,^31^ its pubertal onset and the tight relationship of bone formation by osteoblasts and bone resorption by osteoclasts suggest that osteosarcomagenesis may also be dependent on dysregulation of cellular and hormonal spatiotemporal cues.

Similar to prior studies, we observed a two‐fold increase in ART use among hepatoblastoma patients compared to children with other cancers. This association was not statistically significant given the modest number of hepatoblastoma patients in our dataset (*N* = 61), and the magnitude of effect was greatly attenuated in multivariable models accounting for birthweight. Multiparity and low birthweight were significantly associated with hepatoblastoma risk in univariate analyses, corroborating previous studies.,^8^, ^32^, ^33^, ^34^ but associations were non‐significant in multivariable models adjusting for ART use and gestational age. Notably, we did not observe an association between ART use and rhabdomyosarcoma or ALL, both of which have been previously but not universally observed in prior studies.^25^, ^26^

The proportion of all ART births due to specific ART modalities has shifted over time.^15^ When looking at specific ART modalities in association with hepatoblastoma and osteosarcoma, each ART modality had similar positive magnitudes of effect; however, none reached statistical significance due to the small number of parents who utilized each ART modality. This analysis suggests that no single ART modality drove the overall associations with childhood cancers that were observed. Importantly, IVF was not the only modality with a large odds ratio, nor did it have the largest magnitude of effect across modalities, indicating a need for further studies on the association between ART use and childhood cancer etiology, especially studies that do not focus exclusively on IVF.

Our study has several limitations, including that survey participants were a self‐selected population of caregivers who independently navigated to the ALSF MCC survey portal and who do not represent a random sample of childhood cancer caregivers. A related limitation is that survey respondents are primarily non‐Hispanic white and results may not be broadly generalizable; however, the distribution of household incomes in our sample aligns reasonably well with the income distribution of U.S. households. Our study design compares specific childhood cancer subtypes to all other childhood cancers, rather than to cancer‐free controls, and therefore may attenuate the effect of certain factors that confer risk for multiple cancer subtypes. Due to the self‐selected nature of participants in our study, it would be inappropriate to compare the frequency of ART use in our sample with that in the general population. Although our study design does not permit a “disease‐free” control group for case–control comparisons, our analytic approach comparing specific childhood cancer subtypes to a collapsed “all other cancers” comparator minimizes many of the biases common in case–control study designs, especially recall bias. Parents of children diagnosed at older ages, such as osteosarcoma, may less accurately recall perinatal exposures from many years earlier, although we adjusted for time between birth and survey completion and this did not meaningfully alter results. Our study is relatively large for an epidemiologic assessment of ART and childhood cancer, but several specific cancer types included in analyses were uncommon (e.g. 28 patients with retinoblastoma), limiting precision of our effect estimates. Self‐report biases, including recall bias, may be reduced by utilizing certain study design strategies (e.g., nested case–control designs) and by prospectively enrolling participants. For studies which cannot feasibly enroll prospectively, recall bias can be minimized by recruiting participants with recent diagnoses to shorten recall periods, and by accounting for time elapsed between diagnosis and exposure measurement in the analysis stage using adjustment or stratification. Additionally, self‐report bias may be further reduced by employing high‐quality, validated questionnaires which accurately measure a well‐defined exposure, such as ART use. Another limitation to our study is potential confounding due to the unknown relationship between parental infertility itself and risk of cancer in children, as well as limited information on the presence of birth defects and other congenital abnormalities. Finally, ART information was from self‐report and could not be independently validated, but unlike some registry‐based approaches, our data enabled us to examine associations within strata of specific ART modalities.

Birth registries often capture ART use unreliably and have historically limited data capture to IVF, thus excluding other forms of ART.^35^ Parent‐reported data of ART use has been found to be more reliable overall.^36^ We assessed the effect of ART by childhood cancer subtypes, including stratification by ART modality. Furthermore, because our study did not depend upon registry data, we were able to observe a potential association between ART use and risk of osteosarcoma, a malignancy frequently diagnosed too far into adolescence to be evaluated in studies relying on IVF databases with fewer than 10 years of follow‐up.

Because ART is an increasingly prevalent means of conception, it is important to understand any potential associated risks. Our findings support prior investigations linking ART use to risk of hepatoblastoma, and suggest this association may be attributable to the effect of low birthweight and/or prematurity, an established hepatoblastoma risk factor. Finally, we observed a novel, birthweight‐independent association between ART use and osteosarcoma, emphasizing the need to continue studying ART‐associated cancer risks beyond the first 10 years of life, into adolescence and perhaps continuing throughout adulthood.

## AUTHOR CONTRIBUTIONS

The authors confirm contribution to the paper as follows: *study conception/design, methodology, and investigation*: Gulrajani, Walsh, Landi, Hill, Montes, McGough, Wimberly, Khattab, Semmes, Towry, Hurst, Cohen; *supervision/oversight*: Hill, Towry, Walsh; *funding acquisition*: Walsh; *data curation*: Gulrajani, Hill, Towry, Wimberly; *formal analysis*: Gulrajani; *resources*: Towry, Walsh; *draft manuscript preparation*: Gulrajani. All authors reviewed the results and approved the final version of the manuscript.

## FUNDING INFORMATION

This study was supported by a grant from Alex's Lemonade Stand Foundation (KMW). The funder hosts and advertises the survey portal from which data in this manuscript were collected. The funder had no role in data analysis or interpretation, although an employee of ALSF (LT) is a co‐author of this manuscript in recognition of her significant contributions to constructing and piloting the surveys and her participation in reviewing the final version of the manuscript presented here.

## CONFLICT OF INTEREST

The authors have no conflicts of interest to disclose.

## ETHICS STATEMENT

This study was approved by the Duke University Institutional Review Board (Pro00100771) and did not require informed consent.

## Supporting information


Table S1
Table S2

## Data Availability

De‐identified, individual‐level data are available from the authors upon reasonable request.

## References

[1] Ward E , DeSantis C , Robbins A , Kohler B , Jemal A . Childhood and adolescent cancer statistics, 2014. CA Cancer J Clin. 2014;64(2):83‐103. 24488779 10.3322/caac.21219

[2] Smith MA , Altekruse SF , Adamson PC , Reaman GH , Seibel NL . Declining childhood and adolescent cancer mortality. Cancer. 2014;120(16):2497‐2506. 24853691 10.1002/cncr.28748 PMC4136455

[3] Linabery AM , Ross JA . Trends in childhood cancer incidence in the U.S. (1992‐2004). Cancer. 2008;112(2):416‐432. 18074355 10.1002/cncr.23169

[4] Giddings BM , Whitehead TP , Metayer C , Miller MD . Childhood leukemia incidence in California: high and rising in the Hispanic population. Cancer. 2016;122(18):2867‐2875. 27351365 10.1002/cncr.30129 PMC5542672

[5] Wright E , Amankwah EK , Winesett SP , et al. Incidentally found brain tumors in the pediatric population: a case series and proposed treatment algorithm. J Neurooncol. 2019;141(2):355‐361. 30411179 10.1007/s11060-018-03039-1

[6] Muskens IS , Feng Q , Francis SS , et al. Pediatric glioma and medulloblastoma risk and population demographics: a poisson regression analysis. Neuro‐Oncol Adv. 2020;2(1):vdaa089. 10.1093/noajnl/vdaa089 PMC7447139 32864610

[7] Harvey EB , Boice JD , Honeyman M , Flannery JT . Prenatal x‐ray exposure and childhood cancer in twins. N Engl J Med. 1985;312(9):541‐545. 3969117 10.1056/NEJM198502283120903

[8] O'Neill KA , Murphy MF , Bunch KJ , et al. Infant birthweight and risk of childhood cancer: international population‐based case control studies of 40 000 cases. Int J Epidemiol. 2015;44(1):153‐168. 25626438 10.1093/ije/dyu265

[9] Anderson LM , Diwan BA , Fear NT , Roman E . Critical windows of exposure for children's health: cancer in human epidemiological studies and neoplasms in experimental animal models. Environ Health Perspect. 2000;108(Suppl 3):573‐594. 10852857 10.1289/ehp.00108s3573 PMC1637809

[10] Wiemels JL , Talbäck M , Francis S , Feychting M . Early infection with cytomegalovirus and risk of childhood hematologic malignancies. Cancer Epidemiol Biomark Prev. 2019;28(6):1024‐1027. 10.1158/1055-9965.EPI-19-0044 PMC7731595 30996022

[11] Francis SS , Wallace AD , Wendt GA , et al. In utero cytomegalovirus infection and development of childhood acute lymphoblastic leukemia. Blood 2017;129(12):1680–1684. 27979823 10.1182/blood-2016-07-723148 PMC5364339

[12] Hargreave M , Jensen A , Hansen MK , et al. Association between fertility treatment and cancer risk in children. Jama. 2019;322(22):2203‐2210. 31821431 10.1001/jama.2019.18037 PMC7081748

[13] Spector LG , Brown MB , Wantman E , et al. Association of in Vitro Fertilization with Childhood Cancer in the United States. JAMA Pediatr. 2019;173(6):e190392. 30933244 10.1001/jamapediatrics.2019.0392 PMC6547076

[14] Williams CL , Bunch KJ , Stiller CA , et al. Cancer risk among children born after assisted conception. N Engl J Med. 2013;369(19):1819‐1827. 24195549 10.1056/NEJMoa1301675

[15] State‐Specific Assisted Reproductive Technology Surveillance | CDC . 2020. Available from: https://www.cdc.gov/art/state‐specific‐surveillance/index.html

[16] Helmerhorst FM , Perquin DAM , Donker D , Keirse MJNC . Perinatal outcome of singletons and twins after assisted conception: a systematic review of controlled studies. BMJ. 2004;328(7434):261‐260. 14742347 10.1136/bmj.37957.560278.EE PMC324454

[17] McDonald SD , Han Z , Mulla S , et al. Preterm birth and low birth weight among in vitro fertilization twins: a systematic review and meta‐analyses. Eur J Obstet Gynecol Reprod Biol. 2010;148(2):105‐113. 19833428 10.1016/j.ejogrb.2009.09.019

[18] Davies MJ , Moore VM , Willson KJ , et al. Reproductive technologies and the risk of birth defects. N Engl J Med. 2012;366(19):1803‐1813. 22559061 10.1056/NEJMoa1008095

[19] Maalouf WE , Mincheva MN , Campbell BK , Hardy ICW . Effects of assisted reproductive technologies on human sex ratio at birth. Fertil Steril. 2014;101(5):1321‐1325. 24602756 10.1016/j.fertnstert.2014.01.041

[20] Elias FTS , Weber‐Adrian D , Pudwell J , et al. Neonatal outcomes in singleton pregnancies conceived by fresh or frozen embryo transfer compared to spontaneous conceptions: a systematic review and meta‐analysis. Arch Gynecol Obstet. 2020;302(1):31‐45. 32445067 10.1007/s00404-020-05593-4 PMC7266861

[21] Wimberly CE , Towry L , Caudill C , Johnston EE , Walsh KM . Impacts of COVID‐19 on caregivers of childhood cancer survivors. Pediatr blood cancer. 2021;68(4):e28943 Available from: https://www.ncbi.nlm.nih.gov/pmc/articles/PMC7995053/ 33565259 10.1002/pbc.28943 PMC7995053

[22] Zhang C , Ostrom QT , Hansen HM , et al. European genetic ancestry associated with risk of childhood ependymoma. Neuro‐Oncol 2020;22(11):1637–46. 32607579 10.1093/neuonc/noaa130 PMC7846152

[23] Richiardi L , Askling J , Granath F , Akre O . Body size at birth and adulthood and the risk for germ‐cell testicular cancer. Cancer Epidemiol Biomark Prev. 2003;12(7):669‐673. 12869410

[24] Richiardi L , Akre O , Bellocco R , Ekbom A . Perinatal determinants of germ‐cell testicular cancer in relation to histological subtypes. Br J Cancer. 2002;87(5):545‐550. 12189554 10.1038/sj.bjc.6600470 PMC2376152

[25] Wang T , Chen L , Yang T , et al. Cancer risk among children conceived by fertility treatment. Int J Cancer. 2019;144(12):3001‐3013. 30548591 10.1002/ijc.32062 PMC6590158

[26] Williams CL , Bunch KJ , Murphy MFG , et al. Cancer risk in children born after donor ART. Hum Reprod Oxf Engl. 2018;33(1):140‐146. 10.1093/humrep/dex333 29106578

[27] Zhang C , Morimoto LM , de Smith AJ , et al. Genetic determinants of childhood and adult height associated with osteosarcoma risk. Cancer. 2018;124(18):3742‐3752. 30311632 10.1002/cncr.31645 PMC6214707

[28] Endicott AA , Morimoto LM , Kline CN , Wiemels JL , Metayer C , Walsh KM . Perinatal factors associated with clinical presentation of osteosarcoma in children and adolescents. Pediatr Blood Cancer. 2017;64(6):e26349. 10.1002/pbc.26349 27860191

[29] Tan K , An L , Miao K , et al. Impaired imprinted X chromosome inactivation is responsible for the skewed sex ratio following in vitro fertilization. Proc Natl Acad Sci USA. 2016;113(12):3197‐3202. 26951653 10.1073/pnas.1523538113 PMC4812732

[30] Dan YY , Riehle KJ , Lazaro C , et al. Isolation of multipotent progenitor cells from human fetal liver capable of differentiating into liver and mesenchymal lineages. Proc Natl Acad Sci USA. 2006;103(26):9912‐9917. 16782807 10.1073/pnas.0603824103 PMC1502553

[31] Roberts RD , Lizardo MM , Reed DR , et al. Provocative questions in osteosarcoma basic and translational biology: a report from the Children's oncology group. Cancer. 2019;125(20):3514‐3525. 31355930 10.1002/cncr.32351 PMC6948723

[32] Spector LG , Puumala SE , Carozza SE , et al. Cancer risk among children with very low birth weights. Pediatrics. 2009;124(1):96‐104. 19564288 10.1542/peds.2008-3069 PMC2704984

[33] Spector LG , Johnson KJ , Soler JT , Puumala SE . Perinatal risk factors for hepatoblastoma. Br J Cancer. 2008;98(9):1570‐1573. 18392049 10.1038/sj.bjc.6604335 PMC2391098

[34] Heck JE , Meyers TJ , Lombardi C , et al. Case‐control study of birth characteristics and the risk of hepatoblastoma. Cancer Epidemiol. 2013;37(4):390‐395. 23558166 10.1016/j.canep.2013.03.004 PMC3679264

[35] Luke B , Brown MB , Spector LG . Validation of infertility treatment and assisted reproductive technology use on the birth certificate in eight states. Am J Obstet Gynecol. 2016;215(1):126‐127. 26945609 10.1016/j.ajog.2016.02.052 PMC5257272

[36] Stern JE , McLain AC , Buck Louis GM , Luke B , Yeung EH . Accuracy of self‐reported survey data on assisted reproductive technology treatment parameters and reproductive history. Am J Obstet Gynecol. 2016;215(2):219.e1‐219.e6. 10.1016/j.ajog.2016.02.010 PMC4967378 26875948

