# Supplementary material for: Assisted reproductive technology and association with childhood cancer subtypes
Source: Cancer Med. 2022 Aug 5;12(3):3410–8. doi: 10.1002/cam4.5114 (PMC9939138; doi:10.1002/cam4.5114)
Supplement: Supplementary file 1 — Table S1 Table S2 [file CAM4-12-3410-s001.docx]

| **Supplemental Table 1.** Relationships between ART use, multiparous birth, birthweight and cancer type^a^ | | | | | | |
| --- | --- | --- | --- | --- | --- | --- |
| **Cancer Type** | **ART Use (Odds Ratio)** | **P-value** | **Multiparous Birth (Odds Ratio)** | **P-value** | **Birthweight (1-5 scale)^b^** | **P-value** |
| Hodgkin lymphoma | 1.36 | 0.56 | 1.23 | 0.73 | 2.97 | 0.78 |
| Non-Hodgkin lymphoma | 1.08 | 0.87 | 0.82 | 0.70 | 2.97 | 0.68 |
| Germ cell tumor | 0.46 | 0.43 | 0.89 | 0.87 | 3.05 | 0.08 |
| Wilm's tumor | 0.66 | 0.37 | 0.94 | 0.89 | 2.95 | 0.92 |
| Hepatoblastoma | 2.03 | 0.19 | **2.71** | **0.02** | **2.64** | **<0.001** |
| Neuroblastoma | 0.84 | 0.58 | **1.62** | **0.03** | 2.97 | 0.59 |
| Retinoblastoma | NA | 0.13 | NA | 0.13 | 2.87 | 0.20 |
| Rhabdomyosarcoma | 1.33 | 0.43 | 0.95 | 0.90 | 2.99 | 0.53 |
| Osteosarcoma | **2.56** | **0.01** | 1.68 | 0.23 | 2.96 | 0.83 |
| Ewing sarcoma | 0.63 | 0.43 | 0.42 | 0.22 | 2.97 | 0.72 |
| All sarcomas | 1.40 | 0.15 | 1.17 | 0.54 | 2.96 | 0.96 |
| ALL | 1.11 | 0.76 | 0.73 | 0.45 | 2.97 | 0.31 |
| AML | 0.69 | 0.45 | 0.98 | 0.98 | 2.91 | 0.27 |
| PNET^c^ | 0.89 | 0.77 | 0.83 | 0.64 | 2.98 | 0.52 |
| Ependymoma | 0.92 | 0.92 | 0.40 | 0.34 | 2.90 | 0.62 |
| Astrocytoma^d^ | 1.30 | 0.47 | 1.14 | 0.71 | 2.98 | 0.53 |
| Abbreviations: ART, assisted reproductive technology; PNET, primitive neuroectodermal tumor; AT/RT, atypical teratoid/rhabdoid tumor; DIPG, diffuse intrinsic pontine glioma.  ^a^chi square tests used for ART and multiparous births; t-tests for birthweight; Odds ratios are by subtype, comparing the listed cancer subtype to all other childhood cancer patients in the study. Therefore, it should be noted that the reference group changes slightly for each subtype comparison.  ^b^Mean = 2.96  ^c^Including medulloblastoma and AT/RT  ^d^Including pilocytic, glioblastoma, and DIPG | | | | | | |

| **Supplemental Table 2.** Relationships between ART subtypes and hepatoblastoma and osteosarcoma^a^ | | | | |
| --- | --- | --- | --- | --- |
| **Assisted Reproductive Therapy (ART) Type** | **Hepatoblastoma (Odds Ratio)** | **P-value** | **Osteosarcoma (Odds Ratio)** | **P-value** |
| In vitro fertilization (IVF) | 2.26 | 0.37 | 1.15 | 0.59 |
| Fertility drugs | 2.54 | 0.09 | 1.96 | 0.14 |
| Donor sperm or eggs | NA | - | 3.37 | 0.28 |
| Intracytoplasmic sperm injection (ICSI) | 4.94 | 0.20 | 2.52 | 0.35 |
| Intrauterine insemination (IUI) | NA | - | 2.79 | 0.18 |
| Gamete intrafallopian transfer (GIFT) | NA | - | NA | - |
| ^a^Odds ratios that are NA had 0 observations of specific ART type and cancer diagnosis. P-values shown are Fisher’s exact tests | | | | |
